# SOCIAL HEALTH IN THE CASE OF DEMENTIA: AN INTERNATIONAL PERSPECTIVE

**DOI:** 10.1093/geroni/igac059.1704

**Published:** 2022-12-20

**Authors:** Karin Wolf-Ostermann, Myrra Vernooij-Dassen, Rene Melis

## Abstract

Dementia is one of the major age-related societal challenges and causes enormous demands for persons living with dementia (PlwD) and their families. We do not understand the origins of this multifactorial syndrome and there is still no cure for dementia. New thinking by the exploration of paradigms has scope to improve knowledge about this complex condition. Social health can be understood as a driver for stimulating the use of cognitive reserve through active facilitation and utilization of the individual’s capacities. It allows to slow cognitive impairment or to maintain cognitive functioning in old age and therefore seems to be a promising approach to a better understanding of the developmental mechanism of dementia.In this international symposium we therefore aim to explore the role of social health in the onset and progress of dementia. The first presentation will present a new framework on understanding social health in dementia. The second presentation will describe convoys of care in a family based culturally sensitive ADRD caregiving intervention reducing care burden and family conflicts. The third presentation reports on the role of immune system and neurodegeneration markers in the association between social health and cognitive brain aging in older adults. The final presentation presents newly derived results from a mixed research synthesis on underlying mechanisms of the interrelation of social health and cognitive functioning, elaborated by the international SHARED-consortium. Our discussant will synthesize the research findings and lead a discussion of future directions for research and practice to successfully fight challenges in dementia care.

